# Public awareness of sickle cell disease in Bahrain

**DOI:** 10.4103/0256-4947.65256

**Published:** 2010

**Authors:** Shaikha Al Arrayed, Amani Al Hajeri

**Affiliations:** Salmaniya Medical Complex, Manama, Bahrain

## Abstract

**BACKGROUND AND OBJECTIVES:**

Previous studies that have assessed patient awareness of the management of sickle cell disease (SCD) indicated a lack of awareness of the disease and possibly a need for more public education. Therefore, we measured public awareness in Bahrain of SCD.

**METHODS:**

The study was conducted from December 2006 to February 2007. A questionnaire was distributed among 2000 persons selected from among the general public. The participants had face-to-face interviews with either a health professional or a trained interviewer.

**RESULTS:**

Most (93%) had heard of SCD and 89% knew that it can be diagnosed by a blood test, but 51% did not know the prevalence of SCD in Bahrain. Eighty-four percent recognized it as a hereditary disorder and 72% said that it can skip generations. Females showed better knowledge than males and married persons seems to know more about SCD than unmarried ones.

**CONCLUSION:**

There is a good level of knowledge about SCD among the public, though some of the respondents were confused about the difference between the carrier state of a disease and the disease itself. There is wide acceptance and appreciation of the SCD prevention campaigns being conducted in Bahrain, such as the premarital service and the student screening program.

Genetic diseases, especially hereditary blood disorders such as sickle cell disease (SCD) and the thalassemia syndromes, are a significant problem in many countries. Their chronic nature, with no prospect of cure, make them important causes of morbidity and mortality. The prevalence of hereditary blood diseases in Bahrain is considered high. Previous neonatal screening in 1984-1985 showed that the birth prevalence of SCD was 2.1%, of sickle cell trait 11%, and of glucose-6-phosphate dehydrogenase (G6PD) deficiency 25%.^1^ In 1984, the first genetics clinic was established and several several educational campaigns were started. In 1991, the Bahrain Hereditary Anemia Society was formed. In 1992, the Minister of Health formed a national committee for the prevention of genetic diseases in Bahrain. Screening of all pregnant women began, with testing of the newborn if the mother was found to be a carrier. In 1993, a premarital counseling service was organized and then expanded to include all health centers.^2^ In the year 2004, the Bahrain Government passed a law requiring all couples planning to get married to undergo free premarital counseling.^3^ In 1998 the student screening project began,^4^ and the newborn screening program for blood diseases was launched in 2007.^5^ All these programs were accompanied by educational campaigns that aimed at increasing public awareness about SCD as well as other common hereditary blood disorders.

In 1998, Al Nasir et al conducted a study to assess patient awareness of the management of SCD. Only 30% of the patients in that study were found to have a high degree of knowledge about the disease, and 59% of the subjects thought that there was not enough health education being conducted in the community.^6^ As far as we know, there has been no previous study in Bahrain to measure public awareness of SCD. We measured public awareness of SCD in Bahrain.

## METHODS

The study was conducted from December 2006 to February 2007. A questionnaire was developed to cover three of the commonest inherited blood diseases in Bahrain: SCD, β-thalassemia, and G6PD deficiency. In this report, we present only the results for SCD. Some of the questions about SCD were adapted from the study by Boyd et al.^7^ The questionnaire was distributed among 2000 persons from the general public, including different occupations and ages (school teachers, secondary school students, and others). All participants were interviewed face-to-face either by a health professional or a trained interviewer. The first part of the questionnaire requested personal information such as age, sex, occupation, level of education, and social status. The data was coded and processed using SPSS v 15.0. Frequency tables were obtained and statistical analysis was done using the Mann-Whitney U test (nonparametric test algorithms) and the Kruskal-Wallis one-way analysis of variance (nonparametric test algorithms).

## RESULTS

The response rate was 100%. There were 1106 females (55%) and 894 (45%) males in the study population. While 689 (34.5%) of the respondents were in the age group of 10-19 years, only 15 (0.8%) were older than the age of 30 years (Table 1). Of the respondents 583 were professionals, 406 (20.5%) were students, and 618 (31.3%) were unemployed. There were 966 (48.8%) school students, 900 (45.5%) university graduates, and 92 (4.6%) postgraduates, while 22 (1.1%) respondents were illiterate. One thousand fifty (53%) were single and 932 (47%) were married.

**Table 1:** Age distribution of subjects.

Age (years)	Number	Percent
10-19	689	34.8
20-29	627	31.7
30-39	376	19.0
40-49	213	10.8
50-59	61	3.1
>50	15	0.8
**Total**	**1981**	**100.0**

Total not equal to 2000 due to missing data.

The questionnaire was composed of multiple choice questions and open-ended questions (Tables 2–7). Those who had previously heard of SCD answered 34 items (94%) correctly (*P*<.05). When we tested the relationship between the level of knowledge and gender, the responses were significantly different for 20 questions (*P*<.05). In general, females showed better knowledge of SCD, especially the nature of the disease, the mode of inheritance, its diagnosis, whether they personally had it or not, the difference between SCD and sickle cell trait, and the common symptoms and management. Respondents who were 60 years and older gave more correct answers (*P*<.05). They answered 17 of the 38 items (45%) correctly, which was significantly better than the other age categories (*P*<.05). The age group of 30-39 years was the next best, answering 8 questions (21%) correctly. According to the number of correct responses the order was as follows: 60 years and older > 30-39 years > 40-49 years > 10-29 years and 50-59 years.

**Table 2:** Respondent answers to questions.

	Yes	No	Don’t know
Have you ever heard of SCD?	1856 (93.4)	92 (4.6)	39 (2)
Is SCD a disease of the blood?	1834 (92.8)	34 (1.7)	109 (5.5)
Are there different types of SCD?	1299 (65.4)	146 (7.4)	541 (27.2)
Can SCD be identified by a blood test?	1778 (89)	37 (1.9)	177 (8.9)
What is the prevalence of SCD in Bahrain?	Correct answer (<2%): 44 (2.2)	Incorrect: 928 (46.6)	1019 (51)
What is the prevalence of sickle cell trait in Bahrain?	Correct answer (10%):158 (7.9)	Incorrect: 735 (36.8)	1100 (55.2)
Is SCD an inherited disorder?^a^	1681 (84)	77 (4)	186 (9.6)
Can SCD skip generations?	1421 (72)	179 (9)	384 (19)
Do both parents need to have SCT for a baby to be born with SCD?	1117 (56)	604 (30)	267 (13.4)
If you have SCT could your brother and sister have it too?	854 (43)	894 (45)	242 (12.2)
Is it possible to choose which genes are to be passed on to your children?	493 (25)	966 (49)	525 (26.5)
Are you aware if you are a carrier?	281 (14.2)	Not a carrier:1402 (70.7)	299 (15)
Can SCD effect school performance in children?	1578 (80)	116 (6)	268 (14)
Is there a cure for SCD?	198 (10.1)	1163 (59)	608 (30.9)

Values are n (%). Totals do not add to 2000 because of missing data.

a See Table 5 for specific responses.

**Table 3:** Respondent answers to multiple choice questions.

What conditions may worsen SCD?	Cold weather (71) Hot weather (28) Fatigue (75) Fever (63) Vomiting and diarrhea (39) Lack of air (60)
What is the best way to increase awareness?	Television (51) Health education meetings (35) Written information (14)
Which preventive measures are best?	Premarital checking (97) Health education (97) Enactment of laws (94)

Values are percentages.

**Table 4:** Treatment modalities for sickle cell disease.

	Yes	No	Don’t know	Total
Bed rest	1481 (74.8)	161 (8.1)	339 (17.1)	1981
Oral fluids	1095 (55.4)	290 (14.70)	591 (29.9)	1976
Intravenous saline	1451 (73.1)	128 (6.5)	405 (20.4)	1984
Specific type of food	1172 (59.5)	255 (13.0)	542 (27.5)	1969
Oral analgesia	1341 (67.8)	216 (10.9)	421 (21.3)	1978
Injected analgesia	1359 (68.5)	184 (9.30)	441 (22.2)	1984
Blood transfusion	1053 (53.2)	334 (16.9)	592 (29.9)	1979

Values are n (%).

**Table 5:** Responses to: “How do you get sickle cell disease?”

	Number (%)
You are born with it (It’s hereditary)	1681 (86.5)
You get it from a blood transfusion	58 (3.0)
Food items trigger an attack	58 (3.0)
You can get it some other way (e.g. food, airborne transmission)	19 (1.0)
Don’t know	186 (9.6)
**Total**	**1944 (100.0)**

**Table 6:** Responses to: “Would you say that children with sickle cell disease are more likely to develop the following conditions due to the disease?”

	Number (%)
Pain requiring hospitalization	
Yes	1504 (75.7)
No	157 (7.9)
Don’t know	325 (16.4)
Total	1986 (100.0)
Life threatening infections	
Yes	834 (42.3)
No	450 (22.8)
Don’t know	688 (34.9)
Total	1972 (100.0)
Kidney failure	
Yes	481 (24.5)
No	659 (33.5)
Don’t know	827 (42.0)
Total	1967 (100.0)
Stroke	
Yes	384 (19.6)
No	665 (33.9)
Don’t know	912 (46.5)
**Total**	**1961 (100.0)**

**Table 7:** Responses to: “To what extent do you agree or disagree that sickle cell disease can impact a child’s school performance?”

	Number (%)
Strongly Agree	922 (47.0)
Agree	656 (33.4)
Neither agree nor disagree	268 (13.7)
Disagree	78 (4.0)
Strongly Disagree	38 (1.9)
**Total**	**1962 (100.0)**

Professionals gave the most correct answers. When we tested the relationship between the respondents’ occupation and their level of awareness, the results were as expected: the higher the job status, the more knowledgeable the respondent. Professionals showed a significantly good level of knowledge about the nature of SCD, the different types of SCD, how it is diagnosed, the prevalence of the disease, the inheritance pattern, the symptoms, and the treatment; they were also more likely to know whether they themselves had the disease or not. University students answered 9 of 38 items (24%) correctly, which was significantly better that the performance of respondents with lower level of education (illiterate and school) or higher level of education (postgraduates) (*P*<.05). Twenty-seven of 38 items (71%) were answered correctly by married individuals, which was significantly better than the performance of single individuals (*P*<.05).

## DISCUSSION

On the whole, the respondents had some basic knowledge of SCD. They were aware that SCD could skip a generation and that there are different types of SCD. However, in-depth knowledge about the pattern of inheritance seems to be lacking. The majority did not know the prevalence of the disease or what the carrier status is and how it differs from the disease status.

More than two-thirds of the people included in the study were aware of the commonest symptom of SCD (severe pain). Forty-two percent agreed that it can cause life-threatening infections, 25% agreed that it can cause kidney failure, and 20% agreed that it can lead to stroke, indicating a good level of awareness among the public.

A majority of 80% agreed that SCD can have a negative impact on a child’s school performance, which reflects a good appreciation of the severity of the condition. Upon testing knowledge of aggravating factors, surprisingly, almost two-thirds stated that certain types of food can trigger an attack. In fact, food items do not trigger SCD attacks unless there is accompanying G6PD deficiency, in which case the attack can be triggered by consumption of fava beans. Knowledge of the aggravating factors is fairly good, but more education is needed. Respondents showed good knowledge of the management of SCD crises.

We found that females gave significantly more correct answers than males. This can be attributed to many factors; for example, females receive health education as part of their antenatal care; moreover, in most families they are generally the ones taking care of the family’s health and consequently they tend to be more interested in learning about genetic blood diseases. Based on this finding we recommend more educational campaigns targeting males.

When we analyzed the relationship between level of awareness and age we got unexpected results. We expected older respondents to answer more questions correctly but this was not the case; for example, those in the age-group of 50-59 years gave fewer correct answers than younger people. We also expected that the age-group of 10-19 years would be relatively more knowledgeable, but found instead that they scored the least. Therefore, we believe that integrating the relevant information into the school curriculums is essential.

The respondents’ level of education had an impact on the level of awareness. Undergraduate college students were more aware of hereditary blood disease than postgraduates. This could be attributed to the fact that this category was the target of many national campaigns such as the student screening program that started 10 years ago in 1999. Most of the undergraduates students included in this study were in school when the screening program was implemented. In contrast, most of the postgraduates in our sample were not exposed to these programs, which were implemented after they had left school. Compared to the results reported by Adewuyi, 8 where undergraduates had markedly deficient knowledge regarding the mode of transmission of SCD, the undergraduates in our study had a good knowledge regarding the disease. As expected, married people were better informed than single people. Married couples would have gone through premarital testing and counseling and therefore can be expected to be more knowledgeable about these diseases.

We feel that the student screening program, premarital counseling, and newborn screening service must be continued as these national programs have proved to have tremendous impact. Upon relating the level of awareness and previous knowledge about SCD we found that those who had previously heard of SCD answered more questions correctly.

We feel that rather than relying solely on community seminars and information pamphlets, maximum use should be made of television, which has the potential to be the most effective media to educate the public about SCD. As expected, there is a general agreement that premarital checkups, health education, and legislation are important for increasing awareness of the disease.

We recommend that essential information about common blood diseases, particularly SCD, be included in school curriculums. Informational programs should target the male population, educate the public through TV broadcasts, life lectures, and seminars, emphasize on the nature of inheritance of the common blood diseases, emphasize preventive measures, emphasize the differences between a carrier of an inherited blood disease and an affected individual. More support should be provided to programs that have proven their efficiency (i.e., student screening program, premarital counseling, and newborn screening).

In conclusion, we had an excellent response rate in this study and found a good level of awareness regarding SCD in our study sample. Some of the respondents were confused about the difference between the carrier state of a disease and the affected state. Almost all the respondents support and appreciate the preventive campaigns being conducted in Bahrain such as the premarital service and the student screening program. The most striking and rewarding results were the ones that proved the effectiveness of the screening programs in increasing the awareness among the public about these common hemoglobinopathies, especially SCD.

## References

[1] Al Arrayed S (2005). Campaign to control genetic blood diseases in Bahrain. Community Genet.

[2] Al Arrayed S, Hafadh N, Al-Serafi S (1997). Premarital counselling: An experience from Bahrain. East Mediterr Health J.

[3] Al Arrayed S, Al Hajeri A (2005). Premarital genetic counseling: A new law in the Kingdom of Bahrain. J Health Soc Environ Issues.

[4] Al-Arrayed S, Hafadh N, Amin S, Al-Mukhareq H, Sanad H (2003). Student screening for inherited blood disorders in Bahrain. East Mediterr Health J.

[5] Al Arrayed S, Hamza A, Sultan B, Shome D, Bapat J (2007). Neonatal screening for genetic blood diseases. Bahrain Med Bull.

[6] Al Nasir F, Niazi G (1998). Sickle cell disease: Patients’ awareness and management. Ann Saudi Med.

[7] Boyd JH, Watkins AR, Price CL, Fleming F, DeBaun MR (2005). Inadequate community knowledge about sickle cell disease among African-American women. J Natl Med Assoc.

[8] Adewuyi JO (2000). Knowledge of and attitudes to sickle cell disease and sickle carrier screening among new graduates of Nigerian tertiary educational institutions. Niger Postgrad Med J.

